# Metabolomics of Human Aging and Longevity

**DOI:** 10.1093/geroni/igaf122.2085

**Published:** 2025-12-31

**Authors:** Daniel Evans, Oliver Fiehn, Nicholas Schork, Steven Cummings

**Affiliations:** University of California, San Francisco, San Francisco, California, United States; University of California, Davis, Davis, California, United States; The Translational Genomics Research Institute (TGen), Phoenix, Arizona, United States; California Pacific Medical Center Research Institute, San Francisco, California, United States

## Abstract

Metabolites associated with longevity in a prospective study could lead to predictive longevity biomarkers. The Longevity Consortium (LC) performed a prospective case-cohort metabolomics study of longevity from four cohort studies of elderly individuals: Study of Osteoporotic Fractures (SOF), Osteoporotic Fractures in Men (MrOS), Health, Aging, and Body Composition (Health ABC) Study, and the Cardiovascular Health Study (CHS). Longevity cases were defined by reaching the age corresponding to the 98th survival percentile from United States Social Security Administration life tables. This longevity definition for the 1910 US birth cohort for males is 95 years and for females is 99 years. There were 827 longevity cases and 2,555 members of the subcohort that did not achieve longevity over follow-up. GC-TOF MS was used to measure 322 serum metabolites. Metabolite associations with age were identified with sex-specific regression models adjusted for race, clinic site, study, body mass index (BMI), and estimated glomerular filtration rate (eGFR). The same covariates were included in sex-specific regression models for longevity associations, but age was additionally adjusted for. In a meta-analysis of age-association results from both sexes, older age was associated with lower levels of tryptophan (P = 4x10-5) and higher levels of cystine (P = 4x10-4) and hypotaurine (P = 9x10-3). In a meta-analysis of prospective longevity results from both sexes, higher levels of tryptophan were associated with higher odds of longevity (OR = 2.7, P = 6x10-5), and higher levels of cystine were associated with lower odds of longevity (OR = 0.81, P = 0.02). These metabolites could serve as biomarkers for human aging and longevity.

